# Polycyclic Aromatic Hydrocarbons in Atmospheric PM_2.5_ and PM_10_ of Riyadh City, Saudi Arabia: Levels, Temporal Variation, and Health Impacts

**DOI:** 10.3390/toxics13060424

**Published:** 2025-05-23

**Authors:** Hattan A. Alharbi, Ahmed I. Rushdi, Abdulqader Bazeyad, Khalid F. Al-Mutlaq

**Affiliations:** 1 Department of Plant Protection, College of Food and Agriculture Sciences, King Saud University, P.O. Box 2460, Riyadh 11451, Saudi Arabia; 2 ETAL, 2951 SE Midvale Dr., Corvallis, OR 97333, USA; aimrushdi@gmail.com; 3 College of Earth, Atmospheric, Oceanographic Sciences, Oregon State University, Corvallis, OR 97330, USA

**Keywords:** PAHs, PM_2.5_, PM_10_, Riyadh, air quality, health risks, inhalation

## Abstract

**Background:** Polycyclic aromatic hydrocarbons (PAHs) in atmospheric particulate matter (PM) are high in Saudi cities due to industry and traffic, often exceeding safety limits. This study assesses PM_2.5_ and PM_10_ and health risks in Riyadh’s desert environment. **Method:** High-purity chemicals and PAH standards were used. Air samples were collected at King Saud University, extracted, cleaned, and analyzed by GC-MS. QA/QC ensured accuracy, with RSDs of 4.6–7.9%. **Results:** Seasonal temperature shifts in Riyadh influence PM and PAH levels. Higher summer temperatures raise PM/PAH, posing health risks, especially via inhalation. Winter favors PAH accumulation on particles. **Conclusions:** Seasonal temperature shifts significantly affect PM_2.5_, PM_10_, and PAH levels in Riyadh, with summer posing the highest health risks. Inhalation is the main exposure route, especially for PM_2.5_.

## 1. Introduction

Atmospheric particulate matter (PM), composed of a complex mixture of solid and liquid particles, originates from both natural and anthropogenic sources. Their major sources include dust storms, volcanic eruptions, sea spray, wildfires, combustion processes, industrial emissions, agricultural activities, and residential heating [1,2,3,4,5,6,7,8,9,10]. It is a major contributor of polyclic aromatic hydrocarbons (PAHs) to the environment [11,12,13,14,15,16,17,18]. PAHs are a group of organic compounds composed of multiple aromatic rings. They are released into the atmosphere primarily through the incomplete combustion of organic materials such as coal, oil, gas, wood, and garbage [19,20,21,22,23,24,25,26,27,28,29]. These compounds adhere to fine and coarse PM, facilitating their atmospheric transport and deposition. PAHs are persistent in the environment and bioaccumulate in biosphere and become toxic to aquatic and terrestrial life [30,31,32,33]. The environmental persistence of PAHs and their potential for long-range atmospheric transport contribute to widespread contamination, posing serious environmental and health risks due to their carcinogenic and mutagenic properties) [34,35,36,37]. Prolonged exposure to PAHs, especially through inhalation of polluted air, can lead to respiratory issues [38,39], cardiovascular diseases [40,41], and skin disorders [42,43]. PAHs have been linked to various cancers, including lung, skin, and bladder cancer [39,44,45]. Their ability to induce oxidative stress and damage DNA [46,47] makes them particularly hazardous to human health.

PAHs in the atmospheric particulate matter of the major Saudi Arabian cities, such as Riyadh, Jeddah, and Dhahran, show significant concentrations due to extensive industrial activities and heavy vehicular traffic [9,48,49,50,51,52,53,54]. Riyadh exhibits the highest PM and PAH levels, followed by Jeddah and then Dhahran [10,13,18,51]. Each city’s pollution profile is shaped by distinct sources: traffic and dust in Riyadh, coastal and port activities in Jeddah, and industrial emissions in Dhahran. Research indicates that the average concentration of PAHs in these cities often surpasses international safety thresholds, posing serious environmental and health risks [49,50,51]. The presence of PAHs in the atmosphere is a pressing environmental and public health concern, necessitating constant observing, stringent regulatory measures, and ongoing research to mitigate their impact.

Thus, this research aims to investigate the levels of PM_2.5_ and PM_10_, along with their associated polycyclic aromatic hydrocarbons, and to assess the air quality and public health risks related to both PM and PAHs in Riyadh city, Saudi Arabia. The uniqueness of this study stems from several key aspects, notably its contextual focus, comprehensive analysis, and season-specific health risk assessment conducted in a desert city. Here, extreme climatic conditions and distinctive anthropogenic sources—such as oil-related combustion and dust storms—contribute to air pollution in ways that are not commonly observed or documented in temperate regions.

## 2. Materials and Methods

### 2.1. Standards and Chemicals

All chemicals used were of high purity. A standard mixture of polycyclic aromatic hydrocarbons (PAHs) was obtained from AccuStandard, Inc. (New Haven, CT, USA) with concentration of 2.0 mg/mL in Dichloromethane–Benzene (*v:v*, 50:50). This mixture included the following PAHs: naphthalene (Nap), acenaphthylene (Acy), acenaphthene (Ace), fluorene (Flu), phenanthrene (Phe), anthracene (Ant), fluoranthene (FR), pyrene (Pyr), benzo[a]- anthracene (BaA), chrysene (Chr), benzo[k]fluoranthene (BkF), benzo[a]pyrene (BaP), indeno [1,2,3-cd]pyrene (IDP), dibenz[a,h]anthracene (DBahA), and benzo[ghi]perylene (BghiPye) (Table S1).

### 2.2. Site Description and Sampling Procedure

**Figure 1:**
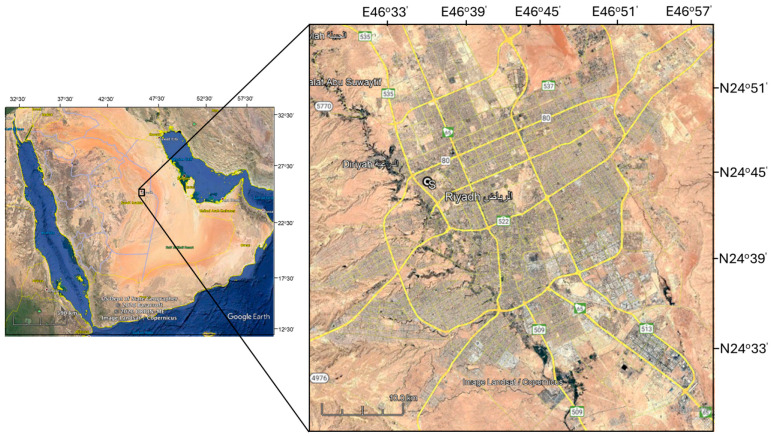
Map showing the locations of Saudi Arabia and the sampling site.

Air samples were collected as described by [55] at the King Saud University (KSU) campus, specifically at the Food and Agriculture Sciences College, located within the Riyadh metropolitan area, which covers approximately 90 km^2^, from April to December 2023. The sampling site (24°43′47.15″ N, 46°36′38.90″ E) is in a remote area, far from any potential air pollution sources, such as roads or industrial areas (Figure 1). Situated northwest of Riyadh, the campus is mainly occupied by university staff, students, and visitors, with a limited number of residential units. This relatively remote area was selected to monitor PM and PAH levels originating from nearby regions, evaluate local human health, and support policy decisions related to air quality and meteorological changes.

Two types of active air sampling devices (from the Tisch Environmental company) were used. A PM_10_ high-volume air sampler was used to collect ambient particulate matter with an aerodynamic diameter of 10 μm or less. This sampler incorporates a size-selective inlet to filter out particles larger than 10 μm, ensuring that only PM_10_ concentrations are deposited onto the filter. These samplers typical operate with a flow rate between 36–60 ft^3^/min during a 24 h sampling period. The PM_2.5_ high-volume air sampler (TE-6001-2.5-I) collects airborne particles smaller than 2.5 microns. These particles are drawn through a size-selective inlet at a flow rate of 40 cubic feet per minute (cfm). The larger particles are trapped within the inlet, while the smaller PM_2.5_ particles pass through and are captured on an 8″ × 10″ glass fiber filter (GFF). All samplers were implemented for a nine-month period, with sampling conducted every two weeks, resulting in a total of 34 sampling events. Typically, prior to installing filters in the sampler, filters were heated in an oven at 550 °C for 12 h overnight and then weighed before and after sampling to determine the mass of collected PM particles. The samplers were calibrated and operated at constant flow rates of 1.4 L/min and for 24 h. Both PM_2.5_ and 10 filters were kept on site, and they were transported in cool boxes at ≈0 °C. All samples were preserved at ≤−18 °C until analysis.

### 2.3. Sample Preparation and Analysis

The sample extraction procedures are described elsewhere [56,57,58], with modifications. Briefly, PM_2.5_ and PM_10_ filters were cut into quarters using sterilized scissors, and one quarter was cut into small pieces (≈1 cm^2^) and inserted into a 50 mL tube. The sample was then sonicated twice using an ultrasonic device (for 30 min) with 2:1 *v*/*v* mixture of dichloromethane and methanol, and then twice with dichloromethane. The internal standards (Naphthalene-d8 and Chrysene-d12) were added to each extract. After extraction, the extract was combined, filtered, and concentrated by rotary evaporator to about 2 mL, and to around 0.5 mL using a gentle stream of high purity nitrogen. For cleanup, the final extract was loaded through the solid phase extraction cartridge (SPE) containing 3 g of silica and eluted with 6 mL of mixture (DCM: Hex 1:1). The eluted solution was concentrated under a gentle stream of N_2_ to dryness, and the final solvent was then changed to high pure hexane.

### 2.4. GC-MS Analysis

The qualitative and quantitative analysis of PAHs was conducted using a gas chromatograph (GC-MS, 6890N Agilent, Palo Alto, CA, USA) equipped with an HP 5973 mass spectrometry detector (MSD). The separation of the PAHs was performed with a DB5-MS capillary column (30 m × 0.25 mm i.d. and film thickness of 0.25 mm, Agilent), with pure helium as the carrier gas at a constant flow rate of 1.3 mL min^−1^. The inlet temperature was set at 280 °C, and the injection was in spitless mode. The oven temperature program consisted of the following sequence: 70 °C and then ramped to 170 °C at rate of 10 °C/min, increased in rate to 15 °C min^−1^, and then to 310 °C, holding for 8 min. The total run time was 27.33 min. MS ion source and MS quadrupole temperatures were maintained at 230 °C and 150 °C, respectively. The MSD was operated in electron impact mode with an ionization energy of 70 eV. The data were acquired and processed with the Agilent ChemStation software (1989–2010 Agilent Technology, Inc.). The identification of the individual compounds was performed by comparison of mass spectra with literature and library data, comparison of mass spectra and GC retention times with those authentic standards and/or interpretation of mass spectrometric fragmentation patterns (Figure S1). The quantification of the individual compounds was using selected ion monitoring (SIM), as listed in Table S1.

### 2.5. Quality Assurance and Quality Control

A rigorous quality assurance and quality control (QA/QC) protocol was implemented to ensure the accuracy and reliability of the analytical data. Method blanks, parallel samples, and solvent blanks were analyzed according to EPA Method 2002 to monitor for contamination and matrix effects. Surrogate standards were added to assess analyte recovery, while internal standards were used to correct for matrix effects and instrument variability. The recovery efficiency for the surrogate standard was 91 ± 13%. A seven-point calibration curve was constructed using PAH-16 standard reagents at concentrations ranging from 1 to 200 ng/mL to quantify the target compounds. To verify the absence of interference and cross-contamination, blank samples and parallel samples were analyzed for each set of samples. A solvent blank and a PAH-16 standard were injected daily to monitor instrument performance. The relative standard deviation (RSD) for all target compounds, determined through replicate injections, ranged from 4.6 to 7.9%, indicating good precision. Final concentrations were corrected based on surrogate recoveries and blank-subtracted.

## 3. Results and Discussion

**Figure 2:**
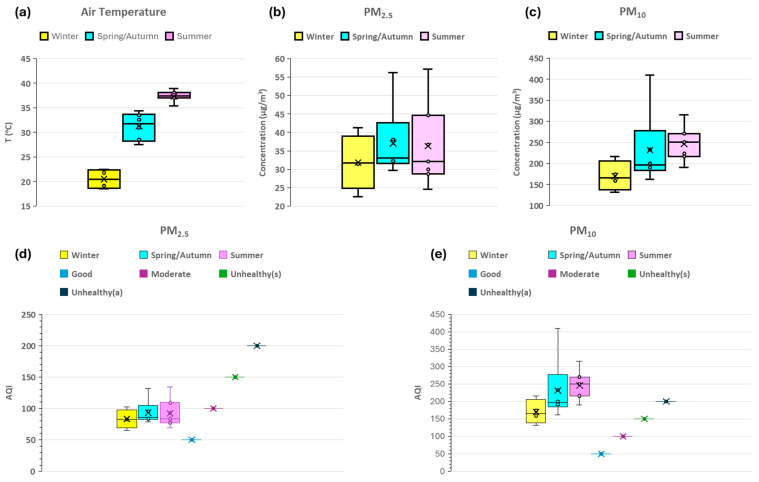
Plots showing: (**a**) the air temperature, (**b**,**c**) PM_2.5_ and PM_10_ concentration and (**d**,**e**) the air quality index (AQI) of PM_2.5_ and PM_10_ ranges during Winter, Spring/Autumn, and Summer for the year 2023 in Riyadh city, Saudi.

Generally, temperature variation influences the formation, dispersion, and concentrations of particulate matter and PAHs in the atmosphere [59,60]. The temperature in Riyadh city showed a clear seasonal pattern (Table 1, Figure 2a), with significantly higher temperatures of 35.4 to 38.9 °C (mean = 37.33 °C) in summer and lower temperatures in winter of 18.5 to 22.5 °C (mean = 20.48 °C). The temperature in spring and autumn ranged between 27.50 to 34.40 °C (mean = 31.25 °C) (Tables S2 and S3). Therefore, the seasons in Saudi Arabia are distinguished by specific temperature thresholds: winter is characterized by temperatures below 25 °C, spring and autumn share a temperature range between 25 °C and 35 °C, and summer begins when temperatures exceed 35 °C. Accordingly, we grouped the results of this study based on these three temperatures ranges (i.e., winter, spring/autumn, and summer).

### 3.1. PM_2.5_ and PM_10_ Concentrations and Air Quality

The detailed discussion of PM_2.5_ and PM_10_ concentration levels can be found in [1]. The concentrations of PM_2.5_ ranged from 22.56 to 41.31 µg/m^3^ 22.56 (mean = 31.85 ± 7.66 µg/m^3^) in winter, 29.69 to 56.19 µg/m^3^ (mean = 37.02 ± 9.78 µg/m^3^) in spring/autumn, and 24.59 to 57.17 µg/m^3^ (mean = 36.27 ± 11.24 µg/m^3^) in summer. PM_10_ concentrations varied from 131.31 to 216.32 µg/m^3^ (mean = 170.07 ± 35.55 µg/m^3^) in winter, 162.13 to 410.36 µg/m^3^ (mean = 231.72 ± 90.39 µg/m^3^) in spring/autumn, and 190.58 to 315.10 µg/m^3^ (mean = 246.07 ± 40.65 µg/m^3^) in summer (Table 1, Figure 2b,c). The data showed higher mean PM_10_ concentrations compared to PM_2.5_ across all seasons, with both exhibiting higher variability in spring/autumn and summer seasons (Figure 2b,c and Figure S2). This variability can be attributed to meteorological factors like temperature inversions, wind pattern, and human activities such as increased energy consumption and vehicular traffic during extreme temperatures [9,60,61]. The PM concentrations, in general, increased with the temperature increase, especially in case of PM_10_, as shown in Figure 2c.

To evaluate the overall PM air quality in Riyadh, we estimated the air quality index (AQI) using the following equation [62]:

AQI_PM_ = [(AQI_H_ − AQI_L_)/(BP_H_ − BP_L_)] × (C_PM_ − BP_L_) + AQI_L_

where BP = break point value, C = concentration of PM, and the subscripts PM = particulate matter, H = high, and L = low. The computed values of AQI for Riyadh, shown in Figure 2d,e, indicate that atmospheric PM_2.5_ concentrations varied from moderate to unhealthy effects for sensitive individuals (i.e., aggravating respiratory conditions such as asthma) [63,64] and contributing to cardiovascular issues [65,66,67]. The levels of PM_10_ effects ranged from unhealthy for sensitive people to unhealthy for all individuals. Increased exposure to PM_10_, coarse particulate matter in the air, has been associated with adverse health effects in both sensitive individuals, exacerbating respiratory conditions such as asthma [68,69], and non-sensitive individuals, contributing to respiratory and cardiovascular morbidity [70,71,72]. Therefore, it is necessary to implement seasonal air quality management strategies, which should include emission controls, urban planning measures, and public health advisories, to reduce the negative effects on public health and environment. The PAH concentrations associated with PM_2.5_ and PM_10_ in Riyadh city across three different seasonal conditions showed variability in their concentrations (Table 1).

### 3.2. PAH Concentrations

The data presented in Table 1 also provide a statistical analysis of the seasonal variations in total polycyclic aromatic hydrocarbon (ΣPAH) concentrations associated with fine (PM_2.5_) and coarse (PM_10_) particulate matter in Riyadh city during the year 2023. The data showed seasonal trends and variability in the concentration levels for both PM_2.5_ and PM_10_. (Figure S3). This variability could be due to fluctuations in emission sources, temperature conditions, and atmospheric chemical process [73,74,75,76]. The total PAH concentrations associated with PM_2.5_ are generally higher than those associated with PM_10_ (Table 1 and Table S1). This suggests that fine particulate matter might have a greater affinity for PAHs or that different sources contribute more significantly to PM_2.5_-associated PAHs [77,78].

During winter (T < 25 °C), the total PAH concentrations in PM_2.5_ ranged from 338.0 ng/g to 639.6 ng/g with a mean of 453.8 ± 144.4 ng/g. The mean concentrations of individual PAHs such as naphthalene (Nap), acenaphthylene (Acy), and chrysene (Chr) were higher compared to those in summer, suggesting that lower temperatures could enhance the condensation of these semi-volatile compounds onto particulate matter. In the transitional seasons of spring and autumn (25 °C–35 °C), the total PAHs concentration varied widely, with significant increase in the mean value (721.1 ng/g). This increase may be due to greater vehicular emissions and industrial activities during these periods, combined with moderate temperatures that allow for both gas–particle partitioning and accumulation of PAHs on particulate matter. The mean concentrations of PAHs like benzo[a]pyrene and benzo[k]fluoranthene were notably higher during this season, indicating potential sources such as traffic emissions and biomass burning [79,80]. The summer season exhibited the highest total PAH concentrations, with values ranging from 590.6 ng/g to 2042.5 ng/g and a mean of 1266.9 ± 536.0 ng/g. The elevated temperatures likely enhance the volatilization of PAHs, but the presence of high concentrations suggests a dominance of PAH emissions from sources such as fossil fuel combustion and vehicular exhausts under high-temperature conditions. The substantial increase in PAHs like benzo[a]pyrene and benzo[k]fluoranthene in summer may be attributed to increased photochemical reaction and high traffic density [81,82,83]. These large molecular weight and more stable compounds resist thermal degradation, remain particle bound, and are typical for intense combustion processes, which account for their higher levels compared to other PAHs.

The total PAH concentrations in PM_10_ during winter ranged from 247.85 ng/g to 566.19 ng/g, with a mean value of 378.18 ± 123.19 ng/g. Similar to PM_2.5_, the winter season favored higher PAH levels in PM_10_, with chrysene and fluoranthene showing significant concentrations. These values were comparable to those reported by [5] but were considerably lower than those documented by [13]. The higher affinity of these PAHs for larger particulate matter could be due to their molecular weight and reduced volatility at lower temperatures [84,85,86]. Total PM_10_ PAH concentration during spring and autumn showed a wide range, with a mean of 398.42 ± 261.48 ng/g. The variability in PAH concentrations during these seasons may reflect changes in atmospheric conditions and emissions sources. Interestingly, the mean concentrations of benzo[a]pyrene increase significantly, indicating an elevated risk of carcinogenic PAHs in the atmosphere during these transitional seasons. The summer season exhibited the lowest total PAH concentrations in PM_10_, with values ranging from 125.15 ng/g to 331.67 ng/g and a mean of 213.92 ± 74.97 ng/g. This reduction in concentration might be due to volatilization of lower molecular weight PAHs at higher temperatures. However, PAHs like benzo[g,h,i]perylene still showed considerable concentration, likely due to persistent sources such as traffic emissions and industrial activities.

The results emphasize the significant seasonal variability in PAH concentrations associated with PM_2.5_ and PM_10_ in Riyadh city. Winter conditions favor higher PAH accumulation due to lower temperatures, while the summer season, despite higher temperatures, shows increased concentrations of specific PAHs, reflecting the influence of source emissions and atmospheric processes [85,86]. The elevated levels of PAHs during transitional seasons, particularly in PM_2.5_, highlight the need for continuous monitoring measures to mitigate the health risks associated with airborne PAHs in urban environments.

### 3.3. PAH Sources

**Figure 3:**
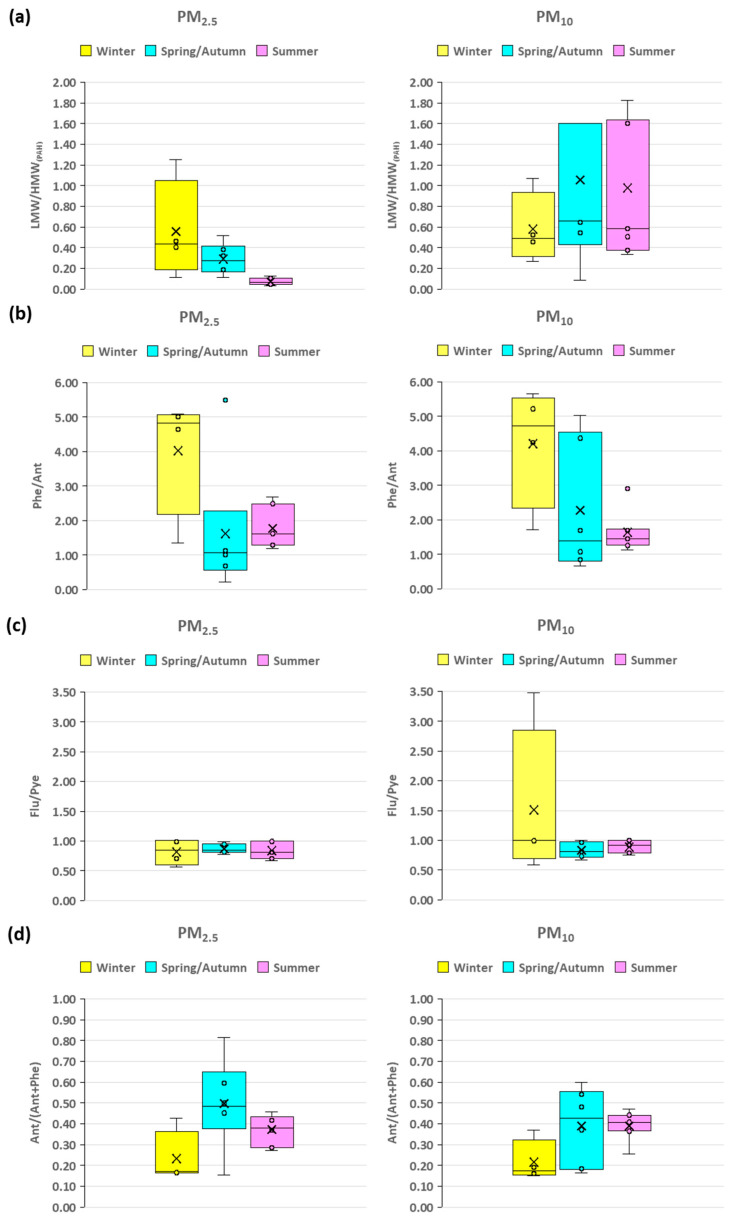

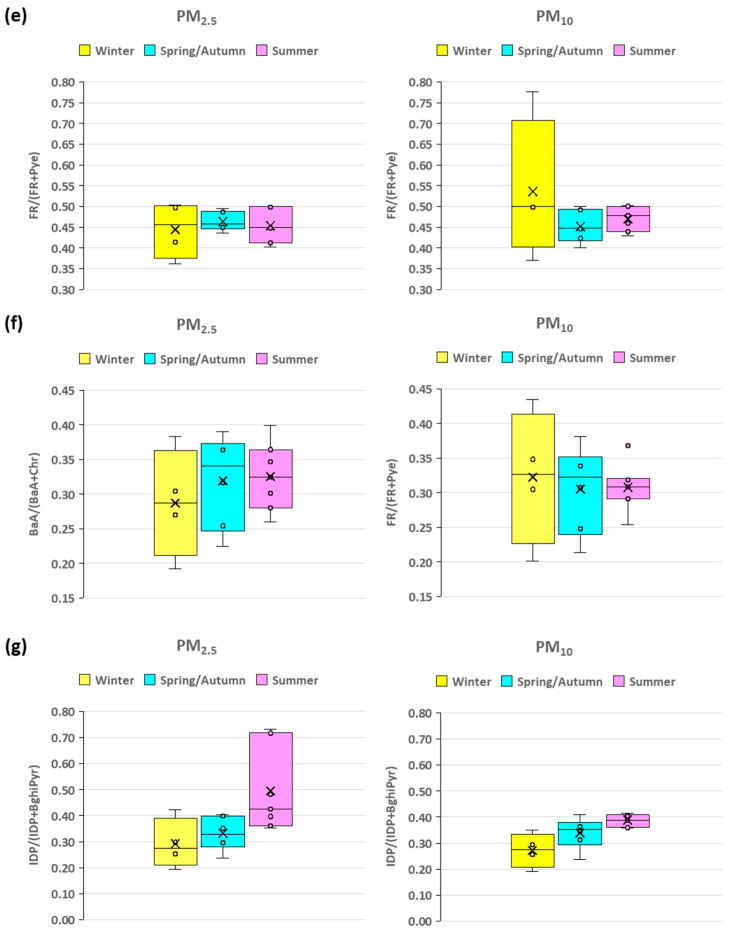
Diagnostic ratio ranges of PM_2.5_ and PM_10_ PAHs: (**a**) LMW/HMW, (**b**) Phe/Ant, (**c**) Flu/Pye, (**d**) Ant/(Ant + Phe), (**e**) FR/(FR + Pyr), (**f**) BaA/(BaA + Chr), (**g**) IDP/(IDP + BghiPyr) during winter, spring/autumn, and summer for the year 2023 in Riyadh city, Saudi.

The PAH source indicators and corresponding diagnostic ratios listed in Table 1 and illustrated in Figure 3 provide insight into their origins across different seasons. The ratios examined include (1) low molecular weight (2–4 rings)/high molecular weight (>4 rings) (LMW/HMW_(PAH)_), (2) phenanthrene/anthracene (Phe/Ant), (3) fluoranthene/pyrene (Flu/Pyr), (4) anthracene/(anthracene + phenanthrene) [Ant/(Ant + Phe)], (5) fluoranthene/(fluoranthene + pyrene) [FR/(FR + Pye)], (6) benzo[a]anthracene/(benzo[a]anthracene + chrysene) [BaA/(AaA + Chr)], and (7) indeno[1,2,3-cd]pyrene/(indeno[1,2,3-cd]pyrene + benzo[ghi]perylene) [IDP/(IDP + BghiPyr)]. These ratios are commonly employed to differentiate between petrogenic sources (derived from crude oil and petroleum products) and pyrogenic sources (derived from combustion processes such as burning of fossil fuels, biomass, or coal)) [87,88,89,90].

The average diagnostic ratios for PM_2.5_ exhibited minimal seasonal variation, with the LHMW/HMW_(PAH)_ ratios consistently below 1 across seasons, indicating a dominant pyrogenic source of PAHs, primarily from combustion processes (Figure 3a). The mean values were higher in winter (0.56) compared to summer (0.07), suggesting that combustion sources such as residential heating contribute more significantly to PAH levels during cooler months. The Phe/Ant ratio remained below 10 throughout all seasons (Figure 3b), with the lowest mean ratio in spring/autumn (1.63), further emphasizing the predominance of pyrogenic sources, likely due to increased combustion activities. The Flu/Pye ratio was close to 1.0 across all seasons and particle sizes (Figure 3c), suggesting a balanced contribution from both petrogenic and pyrogenic sources. However, a slight deviation from 1, particularly in PM_2.5_ during winter (0.82), indicates a predominantly pyrogenic source. During winter, the average Ant/(Ant + Phe) ratio was 0.23 (Figure 3d) suggesting a predominance of combustion sources, as values above 0.1 are typically linked to fuel and coal combustion. This ratio increased significantly to 0.5 in spring/autumn, suggesting a strong influence of combustion processes in these transitional seasons. In summer, the mean ratio decreased to 0.37, still pointing to combustion as a major source. The average FR/(FR + Pye) ratios remained stable between 0.44 and 0.46 across all seasons (Figure 3e), consistently indicating combustion from fuel sources, with low variability (SD ranging from 0.21 to 0.07) implying a stable contribution of sources throughout the year. The average BaA/(BaA + Chr) ratio (Figure 3f) showed a slight increase from winter (0.29) to spring/autumn (0.33), supporting the presence of combustion sources, though with less fluctuation compared to Ant/(Ant + Phe). A significant increase was observed in IDP/(IDP + BghiPyr) ratios (Figure 3g), rising 0.29 in winter to 0.50 in summer, indicating a seasonal shift toward greater fuel combustion during warmer months, possibly due to increased burning conducts.

For PM_10_, the average ratios were comparable to those observed for PM_2.5_. The LMW/HMW_(PAH)_ ratio for PM_10_ (Figure 3a) showed a similar trend to PM_2.5_, generally remaining below 1. However, during spring/autumn, there was a notable increase in the mean ratio (1.06), suggesting a potential mixed contribution from both petrogenic and pyrogenic sources during these transitional seasons. The Phe/Ant ratio is consistently below 10 (Figure 3b), indicating a strong pyrogenic influence. The slight seasonal variation, with a higher mean ratio in winter (4.21), implies that certain conditions, such as lower temperatures, may promote the accumulation of PAHs from combustion. The mean Ant/(Ant + Phe) ratio during winter (0.22; Figure 3d) was comparable to that for PM_2.5_, suggesting combustion sources. In spring/autumn (0.39) and summer (0.39), the mean values were consistent, slightly higher than those in PM_2.5_, possibly due to differences in particulate size affecting the distribution of PAH sources. In winter, the FR/(FR + Py) mean ratio of 0.54 (Figure 3e) indicated some influence from fuel combustion, which was higher than in PM_2.5_, suggesting a potential seasonal shift in the sources contributing to PM_10_. The BaA/(BaA + Chr) ratios (Figure 3f) were slightly lower than PM_2.5_ but still indicated combustion processes, with minimal seasonal variation. The mean IDP/(IDP + BghiPyr) ratio (Figure 3g) in summer (0.39) was higher than in winter (0.27), but lower than in PM_2.5_, suggesting a slightly different source composition in larger particles.

The diagnostic ratios shown in Table 1 and Figure 3 emphasized the dominance of combustion sources throughout all seasons, with distinct seasonal variations suggesting changes in sources contributions. In winter, fuel and combustion were more prevalent, while in summer, there was a significant increase in combustion, particularly in PM_2.5_. These results align with the literature on PAH diagnostic ratios, where similar seasonal patterns have been observed [83,91,92,93].

### 3.4. PAH Health Risk Assessment

Th incremental lifetime cancer risk (*ILCR*) associated with polycyclic aromatic hydrocarbons (PAHs) in PM_2.5_ and PM_10_ via three exposure pathways—inhalation (*ILCR_(Inh)_*), ingestion (*ILCR_(Ing)_*), and dermal absorption (*ILCR_(Der)_*)—is critical for evaluating public health risks, particularly the potential for cancer development due to PAH exposure. They were estimated using the following equations [93,94]:

$${ILCR}_{\left(Inh\right)}=\frac{{BaP}_{eq}\times \left({CSF}_{Inh}\times \sqrt[{3}]{\left(\frac{BW}{70}\right)}\right)\times {IR}_{Inh}\times EF\times ED}{BW\times AT\times PEF}$$

$${ILCR}_{\left(Ing\right)}=\frac{{BaP}_{eq}\times \left({CSF}_{Ing}\times \sqrt[{3}]{\left(\frac{BW}{70}\right)}\right)\times {IR}_{Ing}\times EF\times ED}{BW\times AT\times 10^{6}}$$

$${ILCR}_{\left(Der\right)}=\frac{{BaP}_{eq}\times \left({CSF}_{Der}\times \sqrt[{3}]{\left(\frac{BW}{70}\right)}\right)\times SA\times AF\times ABS\times EF\times ED}{BW\times AT\times 10^{6}}$$

where *IR_Inh_* is the inhalation rate (m^3^ day^−1^), *IR_Ing_* is the soil intake rate (mg day^−1^), *SA* is the dermal surface exposure (cm^2^), *CSF* is carcinogenic slope factor (mg kg^−1^ day^−1^)^−1^, EF is the exposure frequency (day year^−1^), *ED* is the exposure duration (years), *BW* is body weight (kg), AT is the average life span (years), ABS is the dermal adsorption fraction, *PEF* is the particle emission factor (m^3^ kg^−1^), and *AF* is the dermal adherence factor (mg cm^2^ h^−1^). All the parameters used in these models for children (6 years and adults (24 yrs) were based on the Risk Assessment Guidance of the USEPA (2011). Benzo(a)pyrene equivalent (*BaP_eq_*), a value that expresses the overall carcinogenicity of multiple PAHs, was calculated based on the following equation:

$${BaP}_{eq}=\sum C_{i}\times {TEF}_{i}$$

where *C_i_* is the individual concentration of PAHs and *TEF_i_* is the specific values of toxic equivalency factors [95]. The TEFs value of *BaP* is assigned by value 1 and other individual PAHs have different TEFs values based on their carcinogenicity relative to *BaP* [96]. The parameter values are according to [93,97,98].

Table 2 presents a comprehensive assessment of these risks for both children and adults in Riyadh, Saudi Arabia, across different seasons (winter, spring/autumn, and summer) in 2023.

#### 3.4.1. *ILCR* for PM_2.5_

For children, the *ILCR_(Inh)_* value is highest in winter, ranging from 2.40 × 10^−2^ to 6.48 × 10^−2^, with an average of 3.84 × 10^−2^ (Table 2), indicating that inhalation is the primary pathway for cancer risk and falls into the high-risk category (*ILCR* > 10^−3^). In contrast, *ILCR_(Ing)_* and *ILCR_(Der)_* are significantly lower, both falling below 10^−6^, suggesting that ingestion and dermal absorption pose minimal cancer risks in this season. In spring/autumn, inhalation remains the dominant exposure route, with an average *ILCR_(Inh)_* of 2.43 × 10^−2^, although the minimum and maximum values are lower compared to winter, still indicating high risk. *ILCR_(Ing)_* and *ILCR_(Der)_* remain within the minimal risk categories. During summer, the ILCR_(Inh)_ reaches its peak, with values up to 8.67 × 10^−2^ attributed to high-risk levels and extended exposure due to warmer weather. Similarly, *ILCR_(Ing)_* and *ILCR_(Der)_* also increase in summer, reflecting an elevated overall cancer risk for children, although they remain within the small to moderate risk range.

Adults show a pattern similar to children in winter, with inhalation being the primary exposure pathway for cancer risk, as indicated by a mean *ILCR_(Inh_*_)_ of 6.37 × 10^−2^ (Table 2), placing them in the high-risk category. The risks associated with ingestion (*ILCR_(Ing)_*) and dermal absorption (*ILCR_(Der)_*) are much lower, falling within the small risk category, with mean values around 10^−6^. In spring/autumn, the *ILCR_(Inh)_* drops compared to winter, with a mean value of 4.03 × 10^−2^, though it remains in the high-risk category. The reduced PAH concentrations during this cooler season may account for this drop. Ingestion and dermal pathways continue to be less significant, with most values in the small risk range. Similar to children, adults experience a peak in *ILCR_(Inh)_* during summer (mean *ILCR_(Inh)_* = 8.52 × 10^−2^), indicating that inhalation continues to be the primary concern during hotter months. Although *ILCR_(Ing)_* and *ILCR_(Der)_* show higher values in summer compared to other seasons, they still fall within the small-to-moderate risk ranges.

#### 3.4.2. ILCR for PM_10_

For children, the *ILCR_(Inh_*_)_ values for PM_10_ in winter are considerably lower than those for PM_2.5_, with an average of 3.44 × 10^−3^, still indicating a high risk. Both ingestion and dermal pathways contribute minimal risk, with ILCR values below 10^−6^ (Table 2). In spring/autumn, the *ILCR_(Inh_*_)_ values rise (mean ILCR = 8.55 × 10^−3^), reaching the high-risk category for children, likely due to increased particle concentrations during these transitional seasons. Ingestion and dermal pathways remain in the minimal risk category, although the ingestion pathway approaches a higher risk threshold. During summer, the *ILCR_(Inh)_* decreases to a mean of 1.55 × 10^−3^, remaining in the high-risk range, suggesting that PM_10_ concentrations do not increase as sharply as PM_2.5_ during the warmer months. *ILCR_(Ing)_* and *ILCR_(Der_*_)_ remain low risk throughout, with values lower than those observed in other seasons.

Like children, adults show a lower risk from PM_10_ exposure during winter, with a mean *ILCR_(Inh)_* of 5.71 × 10^−3^ (Table 2), classifying them in the high-risk category. The risks associated with ingestion and dermal absorption remain insignificant with values below 10^−6^. However, the inhalation risk significantly increases in spring/autumn, with a mean value of 1.42 × 10^−2^, while the ingestion and dermal exposure continue to pose minimal risks. In summer, *ILCR_(Inh)_* decreases to 2.57 × 10^−3^ (Table 2), still placing it in the high-risk category, and *ILCR_(Ing)_* and *ILCR_(Der)_* remain at low risk.

#### 3.4.3. Total Cancer Risk (*TCR*)

In all seasons and exposure pathways, inhalation remains the primary contributor to total cancer risk (*TCR*), particularly due to PM_2.5_ and PM_10_ particles. The risk is significantly elevated during summer for both adults and children, reflecting the increased pollutant levels during this season. For children, the *TCR* from PM_2.5_ ranges between 2.04 × 10^−3^ and 8.67 × 10^−2^ throughout the year (Table 2), with the highest risk observed in summer (mean = *TCR* = 5.14 × 10^−2^), classified as high risk. Adults show a similar pattern, with their highest mean TCR of 8.52 × 10^−2^, also occurring in summer. In contrast, the *TCR* from PM_10_ is much lower. For children, it ranges from 1.55 × 10^−3^ to 8.55 × 10^−3^, indicating a high risk. Adults show similar trends, with the highest *TCR* in spring/autumn, averaging 1.42 × 10^−2^ (Table 2).

The study’s results are consistent with global research on the cancer risks associated with PAHs in particulate matter. PAHs are known carcinogens, presenting a significant health threat when present in fine particulates (PM_2.5_), especially through inhalation [33,34,99]. Similar high *TCR* values from PAH exposure have been reported in other urban areas with substantial vehicular emissions and industrial activities, such as Basrah, Iraq; Beijing, China; and New Delhi, India [81,100,101,102]. The seasonal variation observed in Riyadh can be explained by meteorological factors like temperature and wind patterns, which affect the dispersion and concentration of pollutants [13,100,103].

## 4. Conclusions

This study assessed the seasonal variations in particulate matter (PM_2.5_ and PM_10_), polycyclic aromatic hydrocarbons (PAHs), and associated health risk in the atmosphere of Riyadh city, Saudi Arabia, during 2023. The findings revealed clear seasonal trends in air temperature, PM levels, PAH concentration, and their sources, with significant implications for urban air quality and public health.

PM_2.5_ and PM_10_ concentrations were generally elevated during the warmer seasons, particularly spring/autumn and summer, influenced by increased energy demand, vehicular activity, and atmospheric dynamics. Air quality index (AQI) values indicated that both PM types often reached levels harmful to sensitive populations and, in some cases, to the general public. These findings underscore the need for seasonal air quality management strategies.

PAH concentrations also displayed distinct seasonal patterns. Total PAHs associated with PM_2.5_ were consistently higher than those in PM_10_, suggesting stronger affinity of fine particles for PAH compounds and a higher contribution from combustion-related sources. Diagnostic ratio analysis confirmed that pyrogenic sources—mainly fossil fuel and biomass combustion—dominate PAH origins across all seasons. However, a slight increase in petrogenic influence was observed in spring/autumn, particularly in PM_10_.

Seasonal health risk assessments based on incremental life cancer risk (ILCR) and total cancer risk (TCR) calculations revealed that inhalation of PAHs via PM_2.5_ in the most critical exposure pathway for both children and adults. ILCR values consistently fell into the high-risk category (ILCR > 10^−3^), especially during summer, when PAH levels and atmospheric reactivity peaked. While ingestion and dermal routes posed relatively lower risks, they still contribute to overall exposure, particularly during hotter months.

Overall, this study highlights the substantial health burden posed by PM-bound PAHs in Riyadh, with clear evidence of seasonal influences on pollution levels and risk magnitude. These results call for urgent, targeted mitigation strategies, including stricter emission regulations, enhanced urban air monitoring, and public health interventions, especially during high-risk periods such as summer and transitional seasons. Long-term integrated air quality policies and public awareness efforts are essential to reduce pollutant exposure and safeguard the health of urban populations in arid and rapidly growing cities like Riyadh.

## Figures and Tables

**Table 1:** Statistical analysis of seasonal (winter, spring/autumn, and summer) atmospheric PM_2.5_ and PM_10_ concentrations, air temperature, PAH concentrations, and diagnostic indices for both particulate matter categories, Riyadh city, Saudi Arabia.

	Winter (T < 25 °C)				Spring/Autumn (25 °C–35 °C)				Summer (T > 35 °C)			
	Min	Max	Mean	SD	Min	Max	Mean	SD	Min	Max	Mean	SD
Avg. Temperature (°C)	18.50	22.50	20.48	1.97	27.50	34.40	31.25	2.77	35.40	38.90	37.33	1.08
	**PM_2.5_**											
PM_2.5_ (mg/m^3^)	22.56	41.31	31.85	7.66	29.69	56.19	37.02	9.78	24.59	57.17	36.27	11.24
AQI	64.89	102.59	83.37	15.40	78.92	131.87	93.48	19.55	68.88	133.80	92.11	22.46
**Compound (ng/g)**												
Naphthalene (Nap)	21.28	57.24	34.32	15.85	5.64	43.24	18.36	16.07	1.15	21.41	8.53	7.15
Acenaphthylene (Acy)	3.35	8.09	5.48	2.38	1.26	8.19	5.80	2.57	5.87	8.41	7.15	0.91
Acenaphthene (Ace)	6.88	139.27	56.19	57.48	6.30	237.04	74.68	95.74	5.44	138.79	40.74	56.85
Fluorene (Flu)	1.58	6.41	3.70	2.03	1.06	7.77	5.62	2.67	4.92	16.47	8.99	4.68
Phenanthrene (Phe)	8.34	30.96	21.46	9.88	4.44	45.24	14.18	15.40	7.77	20.09	12.32	5.21
Anthracene (Ant)	3.95	6.66	5.51	1.19	6.55	30.86	11.91	9.47	5.80	7.66	6.79	0.66
Fluoranthene (FR)	12.73	43.95	25.05	13.53	6.38	62.83	19.17	21.91	6.81	20.89	13.37	5.35
Pyrene (Pyr)	12.58	44.38	31.79	15.28	7.80	63.86	20.80	21.65	8.36	21.84	15.82	5.21
Benz[a]anthracene (BaA)	5.58	21.14	13.82	7.09	5.44	24.21	9.75	7.28	4.83	9.71	7.38	1.64
Chrysene (Chr)	16.86	57.17	34.67	18.19	9.35	70.91	24.23	23.76	7.25	24.93	16.03	5.70
Benz(a)pyrene (BaP)	13.06	260.36	82.15	119.21	−5.06	727.94	253.14	289.83	264.39	989.40	572.34	256.28
Benzo[k]fluoranthene (BkF)	11.80	238.36	74.94	109.30	0.65	657.12	230.20	261.07	241.97	891.28	521.39	227.18
indeno(1,2,3-cd)pyrene (IDP)	7.02	28.01	15.47	8.93	6.18	17.05	9.02	4.16	3.14	48.69	17.73	20.41
Dibenz(a,h)anthracene (DBahA)	2.61	7.32	4.81	2.01	1.51	6.97	4.64	1.90	4.59	12.75	6.88	3.04
Benzo[g,h,i]perylene (BghiPyr)	9.57	82.43	44.45	30.65	9.10	38.29	19.62	11.40	4.77	19.14	11.41	5.26
**Total**	338.0	639.6	453.8	144.4	133.0	1716.5	721.1	593.6	590.6	2041.5	1266.9	536.0
**Diagnostic ratios**												
LMW/HMW_(PAH)_	0.12	1.25	0.56	0.48	0.11	0.52	0.29	0.15	0.03	0.12	0.07	0.03
Phe/Ant	1.35	5.09	4.02	1.80	0.23	5.50	1.63	1.93	1.19	2.69	1.77	0.59
Flu/Pye	0.57	1.01	0.82	0.22	0.77	0.98	0.87	0.08	0.67	1.00	0.84	0.15
Ant/(Ant + Phe)	0.16	0.43	0.23	0.13	0.15	0.81	0.50	0.21	0.27	0.46	0.37	0.07
FR/(FR + Pye)	0.36	0.50	0.44	0.07	0.44	0.50	0.46	0.02	0.40	0.50	0.45	0.05
BaA/(BaA + Chr)	0.19	0.38	0.29	0.08	0.22	0.39	0.32	0.07	0.26	0.40	0.33	0.05
IDP/(IDP + BghiPyr)	0.19	0.42	0.29	0.10	0.24	0.40	0.33	0.07	0.35	0.73	0.50	0.16
	**PM_10_**											
	**Min**	**Max**	**Mean**	**SD**	**Min**	**Max**	**Mean**	**SD**	**Min**	**Max**	**Mean**	**SD**
PM_10_ (mg/m^3^)	131.31	216.32	170.07	35.55	162.13	410.36	231.72	90.39	190.58	315.10	246.07	40.65
AQI	102.74	138.77	120.83	17.00	104.53	280.43	147.81	65.93	118.61	208.15	153.36	31.10
**Compound (ng/g)**												
Naphthalene (Nap)	26.87	47.69	37.25	9.43	5.07	39.68	21.72	15.32	2.07	24.04	9.31	7.88
Acenaphthylene (Acy)	4.55	9.01	6.56	2.04	2.03	8.73	5.95	2.64	6.57	9.30	7.58	0.92
Acenaphthene (Ace)	8.69	119.85	46.59	50.01	7.65	228.59	85.98	92.28	4.99	121.59	47.69	51.81
Fluorene (Flu)	2.21	4.93	3.50	1.23	1.36	9.98	6.02	3.56	4.98	17.66	9.54	5.29
Phenanthrene (Phe)	12.98	29.95	23.89	7.65	4.31	30.30	13.23	9.71	8.32	33.11	15.25	8.16
Anthracene (Ant)	4.66	7.57	6.07	1.45	3.48	9.63	6.57	1.98	7.43	11.36	8.94	1.39
Fluoranthene (FR)	22.22	67.61	35.76	21.34	6.42	28.95	16.35	9.90	11.49	40.15	18.25	9.82
Pyrene (Pyr)	7.77	67.98	35.59	26.35	7.94	43.23	20.37	13.68	14.36	40.01	20.24	9.24
Benz[a]anthracene (BaA)	10.06	51.08	24.27	18.27	5.28	15.21	9.47	4.60	7.50	19.64	10.49	4.23
Chrysene (Chr)	31.95	66.31	45.30	14.76	8.56	46.07	24.21	15.50	17.02	57.66	24.79	14.69
Benz(a)pyrene (BaP)	12.81	43.23	24.95	13.00	3.99	393.97	77.11	155.54	1.49	22.50	8.59	6.56
Benzo[k]fluoranthene (BkF)	16.66	38.82	23.72	10.25	0.94	358.02	69.10	141.90	0.28	25.34	5.09	9.02
indeno(1,2,3-cd)pyrene (IDP)	10.99	26.74	15.42	7.57	6.33	27.67	11.76	7.92	6.99	13.08	8.79	2.00
Dibenz(a,h)anthracene (DBahA)	3.07	7.35	4.87	2.11	2.88	8.88	5.49	1.97	4.48	6.34	5.27	0.59
Benzo[g,h,i]perylene (BghiPyr)	21.95	78.12	44.42	25.87	11.12	60.97	25.11	19.61	9.87	23.52	14.11	4.55
**Total**	247.85	566.19	378.18	134.19	117.04	874.38	398.42	261.48	125.15	331.67	213.92	74.97
**Diagnostic ratios**												
LMW/HMW_(PAH)_	0.27	1.07	0.58	0.35	0.09	3.39	1.06	1.18	0.34	1.82	0.98	0.67
Phe/Ant	1.71	5.65	4.21	1.76	0.67	5.03	2.28	1.92	1.12	2.91	1.65	0.60
Flu/Pye	0.59	3.47	1.52	1.32	0.67	1.00	0.83	0.13	0.75	1.01	0.89	0.10
Ant/(Ant + Phe)	0.15	0.37	0.22	0.10	0.17	0.60	0.39	0.18	0.26	0.47	0.39	0.07
FR/(FR + Pye)	0.37	0.78	0.54	0.17	0.40	0.50	0.45	0.04	0.43	0.50	0.47	0.03
BaA/(BaA + Chr)	0.20	0.44	0.32	0.10	0.21	0.38	0.31	0.06	0.25	0.37	0.31	0.03
IDP/(IDP + BghiPyr)	0.19	0.35	0.27	0.07	0.24	0.41	0.34	0.06	0.36	0.41	0.39	0.02

LMW/HMW_(PAH)_ ratio < 1 = pyrogenic (combustion), >1 = petrogenic (petroleum); Phe/Ant ratio < 10 = pyrogenic (combustion), >10 = petrogenic (petroleum); Flu/Pyr ratio < 1.0 = petrogenic (petroleum), >1 = pyrogenic (combustion); Ant/(Ant + Phe) ratio < 0.1 = petrogenic source, >0.1 = pyrogenic (combustion from fuel and coal); Flu/(Flu + Pyr) ratio < 0.4 = petrogenic sources, 0.4–0.5 = combustion from fuel, >0.5 = combustion from coal and wood; BaA/(BaA + Chr) ratio < 0.2 = petrogenic sources, 0.2–0.5 = fossil fuel combustion >0.50 combustion (grass, wood, coal); Ind/(Ind + BghiP) ratio < 0.2 = petrogenic sources, 0.20–0.5 combustion (fuel), >0.5 combustion from coal and wood.

**Table 2:** The incremental lifetime cancer risk (*ILCR*) and total cancer risk (*TCR*) of atmospheric PAHs in PM_2.5_ and PM_10_ from Riyadh city, Saudi Arabia, through the three pathways: ingestion (*ILCR_(Ing)_*), inhalation (*ILCR_(Inh)_*), and dermal (*ILCR_(Der)_*), during winter, spring/autumn, and summer 2023.

	**PM2.5**											
	**Winter (T < 25 °C)**				**Spring/Autumn (25 °C–35 °C)**				**Summer (T > 35 °C)**			
	**Min**	**Max**	**Mean**	**SD**	**Min**	**Max**	**Mean**	**SD**	**Min**	**Max**	**Mean**	**SD**
	**Children**											
BaP_eq_	0.26	0.70	0.41	0.20	0.02	0.70	0.26	0.26	0.26	0.94	0.55	0.24
ILCR_(Ing)_	1.24 × 10^−6^	3.34 × 10^−6^	1.98 × 10^−6^	9.79 × 10^−7^	1.05 × 10^−7^	3.34 × 10^−6^	1.25 × 10^−6^	1.26 × 10^−6^	1.24 × 10^−6^	4.47 × 10^−6^	2.65 × 10^−6^	1.14 × 10^−6^
ILCR_(Inh)_	2.40 × 10^−2^	6.48 × 10^−2^	3.84 × 10^−2^	1.90 × 10^−2^	2.04 × 10^−3^	6.48 × 10^−2^	2.43 × 10^−2^	2.44 × 10^−2^	2.41 × 10^−2^	8.67 × 10^−2^	5.14 × 10^−2^	2.21 × 10^−2^
ILCR_(Der)_	1.55 × 10^−6^	4.17 × 10^−6^	2.47 × 10^−6^	1.22 × 10^−6^	1.31 × 10^−7^	4.17 × 10^−6^	1.56 × 10^−6^	1.57 × 10^−6^	1.55 × 10^−6^	5.58 × 10^−6^	3.31 × 10^−6^	1.42 × 10^−6^
TCR	2.40 × 10^−2^	6.48 × 10^−2^	3.84 × 10^−2^	1.90 × 10^−2^	2.04 × 10^−2^	6.48 × 10^−2^	2.43 × 10^−2^	2.44 × 10^−2^	2.41 × 10^−2^	8.67 × 10^−2^	5.14 × 10^−2^	2.21 × 10^−2^
	**Adults**											
ILCR_(Ing)_	2.05 × 10^−6^	5.54 × 10^−6^	3.28 × 10^−6^	1.62 × 10^−6^	1.74 × 10^−7^	5.54 × 10^−6^	2.08 × 10^−6^	2.08 × 10^−6^	2.06 × 10^−6^	7.41 × 10^−6^	4.39 × 10^−6^	1.89 × 10^−6^
ILCR_(Inh)_	3.98 × 10^−2^	1.07 × 10^−1^	6.37 × 10^−2^	3.15 × 10^−2^	3.38 × 10^−3^	1.07 × 10^−1^	4.03 × 10^−2^	4.04 × 10^−2^	4.00 × 10^−2^	1.44 × 10^−2^	8.52 × 10^−2^	3.67 × 10^−2^
ILCR_(Der)_	1.82 × 10^−6^	4.92 × 10^−6^	2.92 × 10^−6^	1.44 × 10^−6^	1.55 × 10^−6^	4.92 × 10^−6^	1.84 × 10^−6^	1.85 × 10^−6^	1.83 × 10^−6^	6.58 × 10^−6^	3.90 × 10^−6^	1.68 × 10^−6^
TCR	3.98 × 10^−2^	1.07 × 10^−1^	6.37 × 10^−2^	3.15 × 10^−2^	3.38 × 10^−3^	1.07 × 10^−1^	4.03 × 10^−2^	4.04 × 10^−2^	4.00 × 10^−2^	1.44 × 10^−1^	8.52 × 10^−2^	3.67 × 10^−2^
	**PM10**											
	**Winter (T < 25 °C)**				**Spring/Autumn (25 °C–35 °C)**				**Summer (T > 35 °C)**			
	**Min**	**Max**	**Mean**	**SD**	**Min**	**Max**	**Mean**	**SD**	**Min**	**Max**	**Mean**	**SD**
	**Children**											
BaP_eq_	0.02	0.06	0.04	0.02	0.01	0.44	0.09	0.17	0.01	0.04	0.02	0.01
ILCR_(Ing)_	1.14 × 10^−7^	3.05 × 10^−7^	1.78 × 10^−7^	8.65 × 10^−8^	6.02 × 10^−8^	2.09 × 10^−6^	4.41 × 10^−7^	8.11 × 10^−7^	4.35 × 10^−8^	1.70 × 10^−7^	8.00 × 10^−8^	4.12 × 10^−8^
ILCR_(Inh)_	2.21 × 10^−3^	5.92 × 10^−3^	3.44 × 10^−3^	1.68 × 10^−3^	1.17 × 10^−3^	4.05 × 10^−3^	8.55 × 10^−3^	1.57 × 10^−2^	8.44 × 10^−4^	3.30 × 10^−3^	1.55 × 10^−3^	7.99 × 10^−4^
ILCR_(Der)_	1.42 × 10^−7^	3.81 × 10^−7^	2.21 × 10^−7^	1.08 × 10^−7^	7.51 × 10^−8^	2.61 × 10^−6^	5.49 × 10^−7^	1.01 × 10^−6^	5.42 × 10^−8^	2.12 × 10^−7^	9.97 × 10^−8^	5.14 × 10^−8^
TCR	2.21 × 10^−3^	5.92 × 10^−3^	3.44 × 10^−3^	1.68 × 10^−3^	1.17 × 10^−3^	4.05 × 10^−2^	8.55 × 10^−3^	1.57 × 10^−2^	8.44 × 10^−4^	3.30 × 10^−3^	1.55 × 10^−3^	7.99 × 10^−4^
	**Adults**											
ILCR_(Ing)_	1.89 × 10^−7^	5.06 × 10^−7^	2.94 × 10^−7^	1.43 × 10^−7^	9.98 × 10^−8^	3.46 × 10^−6^	7.30 × 10^−7^	1.34 × 10^−6^	7.21 × 10^−8^	2.82 × 10^−7^	1.33 × 10^−7^	6.83 × 10^−8^
ILCR_(Inh)_	3.66 × 10^−3^	9.81 × 10^−3^	5.71 × 10^−3^	2.78 × 10^−3^	1.94 × 10^−3^	6.72 × 10^−2^	1.42 × 10^−2^	2.60 × 10^−2^	1.40 × 10^−3^	5.46 × 10^−3^	2.57 × 10^−3^	1.32 × 10^−3^
ILCR_(Der)_	1.68 × 10^−7^	4.49 × 10^−7^	2.61 × 10^−7^	1.27 × 10^−7^	8.87 × 10^−8^	3.08 × 10^−6^	6.49 × 10^−7^	1.19 × 10^−6^	6.40 × 10^−8^	2.50 × 10^−7^	1.18 × 10^−7^	6.07 × 10^−8^
TCR	3.66 × 10^−3^	9.81 × 10^−3^	5.71 × 10^−3^	2.78 × 10^−3^	1.94 × 10^−3^	6.72 × 10^−2^	1.42 × 10^−2^	2.61 × 10^−2^	1.40 × 10^−3^	5.46 × 10^−3^	2.57 × 10^−3^	1.32 × 10^−3^

ILCR < 10^−6^ = min risk, ILCR > 10^−6^–< 10^−4^ = small risk, ILCR > 10^−4^–< 10^−3^ = moderate risk, ILCR > 10^−3^–< 10^−1^ = high risk, and ILCR > 10^−1^ = very high risk.

## Data Availability

The original contributions presented in this study are included in the article. Further inquiries can be directed to the corresponding author.

## Supplementary Materials

The following supporting information can be downloaded at: https://www.mdpi.com/article/10.3390/toxics13060424/s1, Table S1: Parameters used in the SIM mode to identify and quantify the individual PAH compounds in the atmospheric PM_2.5_ and PM_10_ samples. Table S2: Meteorological conditions of Riyadh city during sample acquisition. Table S3: The average air temperatures (°C), atmospheric PM_2.5_ and PM_10_ concentrations (mg/g), and levels of different polycyclic aromatic hydrocarbons (PAHs, (ng/g)) of the Riyadh city, Saudi Arabia from April–December 2023. Table S4: Diagnostic ratios of PAHs in atmospheric PM_2.5_ and PM_10_ from Riyadh city, Saudi Arabia from April–December 2023. Figure S1: TIC of the standard mixture of polycyclic aromatic hydrocarbons (PAHs), including the followings: (1) naphthalene (Nap), (2) acenaphthylene (Acy), (3) acenaphthene (Ace), (4) fluorene (Flu), (5) phenanthrene (Phe), (6) anthracene (Ant), (7) fluoranthene (FR), (8) pyrene (Pyr), (9) benzo[a]anthracene (BaA), (10) chrysene (Chr), (11) benzo[b]fluoranthene (BbF), (12) benzo[k]fluoranthene (BkF), (13) benzo[a]pyrene (BaP), (14) indeno[1,2,3-cd]pyrene (IDP), and (15) dibenz[a,h]anthracene (DBahA). Figure S2: Plots showing (a) the concentrations, (b) the concentration ratios of PM_2.5_/PM_10_, and (c) the air quality index (AQI) of atmospheric PM_2.5_ and PM_10_ from the city of Riyadh, Saudi Arabia during the months of April–December of 2023. Figure S3: Box plots showing: (a) and (b) the total concentrations of PAHs in atmospheric PM_2.5_ and PM_10_, respectively, and (c) the total concentration ratios of PAHs in PM_2.5_ to PM_10_ from Riyadh city during the months of April–December of 2023.
