# Supplementary material for: Polycyclic Aromatic Hydrocarbons in Atmospheric PM2.5 and PM10 of Riyadh City, Saudi Arabia: Levels, Temporal Variation, and Health Impacts
Source: Toxics. 2025 May 23;13(6):424. doi: 10.3390/toxics13060424 (PMC12197016; doi:10.3390/toxics13060424)

Supplementary Materials

Polycyclic Aromatic Hydrocarbons in Atmospheric $PM_{2.5}$ and PM_{10} of Riyadh City, Saudi Arabia: Levels, Temporal Variation, and Health Impacts

Hattan A. Alharbi [1,*], Ahmed I. Rushdi [2,3], Abdulqader Bazeyad [1] and Khalid F. Al-Mutlaq [1]

[1] Department of Plant Protection, College of Food and Agriculture Sciences, King Saud University,
P.O. Box 2460, Riyadh 11451, Saudi Arabia; abazeyad@ksu.edu.sa (A.B.); almutlaqk@gmail.com (K.F.A.-M.)

[2] ETAL, 2951 SE Midvale Dr., Corvallis, OR 97333, USA; aimrushdi@gmail.com

[3] College of Earth, Atmospheric, Oceanographic Sciences, Oregon State University, Corvallis, OR 97330, USA

* Correspondence: halharbii@ksu.edu.sa

Hattan A. Alharbi (halharbii@ksu.edu.sa) ORCID: 0000-0003-3297-729 X

Ahmed I. Rushdi (aimrushdi@gmail.com) ORCID: 0000-0002-5967-8620

Abdulqader Bazeyad (abazeyad@ksu.edu.sa) ORCID: 0000-0003-0463-9429

Khalid F. Al-Mutlaq (almutlaqk@gmail.com)

*Corresponding author: Hattan A. Alharbi (halharbii@ksu.edu.sa)

Table S1. Parameters used in the SIM mode to identify and quantify the individual PAH compounds in the atmospheric $PM_{2.5}$ and PM_{10} samples.

#	Compound	Rt	Target ion	Q1	Q2	Q3	Recovery %
1	Naphthalene	7.02	128	136	127		92.56
2	Acenaphthylene	10.58	152	151	150		92.86
3	Acenaphthene	10.99	153	154	152		96.67
4	Fluorene	12.05	166	165	163		100.54
5	Phenanthrene	13.88	178	176	179		98.13
6	Anthracene	13.96	178	176	179		98.80
7	Fluoranthene	15.98	202	200	203		99.51
8	Pyrene	16.36	202	200	203		100.27
9	Benz[a]anthracene	18.36	228	229	226		95.08
10	Chrysene	18.43	228	226	229		102.59
11	Benz(a)pyrene	20.07	252	253	250	251	93.51
12	Benzo[k]fluoranthene	20.11	252	253	250	126	100.45
13	indeno(1,2,3-cd) pyrene	22.93	276	274	275		95.13
14	Dibenz(a,h)anthracene	22.96	278	279	276		93.06
15	Benzo[g,h,i]perylene	23.58	276	138	277	274	95.62

Table S2. Meteorological conditions of Riyadh city during sample acquisition.

Month	Day	T	TM	Tm	SLP	H	PP	VV	V	VM	VG
**April**	4	22	30.0	13.0	1015.9	27	0	9.8	4.4	14.8	-
	25	28.2	34.3	21.0	1008.1	31	0	8.5	12	24.1	-
**May**	10	33.7	40.0	27.0	1005.5	21	0	9.7	9.8	20.6	-
	25	32.4	38.1	26.6	1007.8	28	0	9.7	9.1	20.6	-
**June**	10	35.7	42.0	26.0	1001.9	9	0	10	6.9	18.3	-
	25	37.3	44.0	28.6	999.1	7	0	9.7	11.1	29.4	-
**July**	10	38.9	46.4	30.0	997.7	6	0	10	8	22.2	-
	25	37.4	45.3	27.0	997.2	7	0	9.3	4.8	9.4	-
**August**	10	36.7	44.4	28.0	1001.5	8	0	10	13	25.9	-
	25	37.9	45.1	30.0	1000.0	7	0	10	10.2	20.6	44.6
**September**	10	36.9	44.0	30.0	1002.0	9	0	9.7	6.3	14.8	-
	29	32.6	40	22	1008.9	9	0	9.8	7	24.1	-
**October**	10	30.7	38.0	22.0	1011.9	14	0	9.8	4.6	11.1	-
	25	27.4	33.0	21.0	1013.7	35	0	9.7	10	31.7	-
**November**	10	23.1	29.0	14.7	1018.4	26	0	9.8	3.9	9.4	-
	25	19.5	24.1	15.0	1016.4	68	0	8.2	10	13	-
**December**	10	18.1	27.0	9.5	1019.6	21	0	10	2.6	7.6	-
											-

T =Average Temperature (°C), TM=Maximum temperature (°C), Tm= Minimum temperature (°C), SLP= Atmospheric pressure at sea level (hPa), H=Average relative humidity (%), PP = Total rainfall and / or snowmelt (mm), VV=Average visibility (Km), V=Average wind speed (Km/h), VM=Maximum sustained wind speed (Km/h),VG=Maximum speed of wind (Km/h)

Table S3. The average air temperatures (° C), atmospheric $PM_{2.5}$ and PM_{10} concentrations (mg/g), and levels of different polycyclic aromatic hydrocarbons (PAHs, (ng/g)) of the Riyadh city, Saudi Arabia from April- December 2023.

	**PM2.5 (concentration ng/g)**																
	**4-Apr**	**25-Apr**	**10-May**	**25-May**	**10-Jun**	**25-Jun**	**10-Jul**	**25-Jul**	**10-Aug**	**25-Aug**	**10-Sep**	**25-Sep**	**10-Oct**	**25-Oct**	**10-Nov**	**25-Nov**	**11-Dec**
**Avg. Temperature (°C)**	21.80	28.50	34.40	32.60	35.40	37.50	38.90	37.40	37.00	38.10	37.00	33.50	31.00	27.50	22.50	19.10	18.50
**PM2.5 (mg/g)**	32.03	29.69	32.21	32.97	28.78	44.66	32.12	24.59	36.69	57.17	29.90	33.00	38.04	56.19	41.31	22.56	31.49
**AQI (PM2.5)**	83.53	78.915	83.888	85.387	77.125	109.18	83.708	68.881	92.704	133.8	79.347	85.442	95.354	131.87	102.59	64.893	82.466
**Compound (ng/g)**																	
**Naphthalene**	31.59	33.19	6.50	5.64	7.52	13.98	1.35	21.41	1.15	7.74	6.59	6.57	43.24	15.01	21.28	27.18	57.24
**Acenaphthylene**	6.91	7.29	6.77	8.19	6.53	7.58	8.09	8.41	5.87	6.76	6.85	6.94	4.33	1.26	3.35	3.59	8.09
**Acenaphthene**	6.88	6.30	147.61	237.04	106.84	138.79	11.77	8.24	7.49	5.44	6.61	7.82	24.60	24.68	139.27	41.54	37.08
**Fluorene**	6.41	6.15	7.77	7.18	5.14	5.99	16.47	14.47	9.20	4.92	6.71	7.72	3.83	1.06	1.58	3.02	3.80
**Phenanthrene**	8.34	4.44	7.09	7.72	8.15	11.44	20.09	19.09	8.13	7.77	11.54	8.86	45.24	11.70	19.79	26.75	30.96
**Anthracene**	6.19	6.55	30.86	6.81	6.28	7.05	7.47	7.66	5.80	6.52	6.78	7.32	8.23	11.69	3.95	5.25	6.66
**Fluoranthene**	12.73	6.85	7.83	6.38	10.57	19.56	20.89	15.46	6.81	9.44	10.90	12.01	62.83	19.12	18.70	24.82	43.95
**Pyrene**	12.58	8.14	9.28	7.80	10.55	19.48	20.98	21.84	8.36	14.03	15.50	15.55	63.86	20.16	26.42	43.80	44.38
**Benz[a]anthracene**	10.47	5.44	5.78	6.13	6.13	8.08	6.95	9.71	4.83	8.77	7.21	10.09	24.21	6.86	5.58	18.08	21.14
**Chrysene**	16.86	9.35	10.08	9.56	12.74	18.70	19.76	24.93	7.25	15.29	13.57	21.79	70.91	23.70	23.44	41.23	57.17
**Benz(a)pyrene**	260.36	274.24	443.00	727.94	618.72	705.75	989.40	695.04	264.39	419.43	313.69	-5.06	40.29	38.43	13.06	18.94	36.23
**Benzo[k]fluoranthene**	238.36	250.80	401.93	657.12	559.30	637.24	891.28	627.66	241.97	406.17	286.12	0.65	36.19	34.52	11.80	17.07	32.55
**indeno(1,2,3-cd)pyrene**	7.02	6.28	6.18	6.53	46.37	48.69	6.27	7.16	5.82	3.14	6.63	9.47	8.59	17.05	14.43	12.44	28.01
**Dibenz(a,h)anthracene**	5.35	5.29	4.63	6.97	12.75	4.66	9.22	4.59	5.72	6.23	4.98	5.81	1.51	3.64	3.99	2.61	7.32
**Benzo[g,h,i]perylene**	9.57	9.51	9.10	15.52	16.84	19.14	11.46	12.70	7.84	4.77	7.11	17.49	27.84	38.29	34.10	51.68	82.43
**Total**	639.61	639.84	1104.42	1716.53	1434.41	1666.13	2041.46	1498.35	590.62	926.42	710.79	133.01	465.71	267.16	340.71	337.99	496.99

	**PM10**																
	**(concentration ng/g)**																
	**4-Apr**	**25-Apr**	**10-May**	**25-May**	**10-Jun**	**25-Jun**	**10-Jul**	**25-Jul**	**10-Aug**	**25-Aug**	**10-Sep**	**25-Sep**	**10-Oct**	**25-Oct**	**10-Nov**	**25-Nov**	**11-Dec**
**PM10 (mg/g)**	174.13	200.15	191.01	193.78	315.10	270.47	216.40	190.58	224.02	250.35	255.53	162.13	232.87	410.36	131.31	216.32	158.51
**AQI (PM10)**	110.5	123.3	118.8	120.2	180.7	208.2	131.4	118.6	135.2	148.2	151.3	104.5	139.5	280.4	138.8	131.4	102.7
**Compound (ng/g)**																	
**Naphthalene**	42.22	37.18	5.92	5.07	10.53	12.93	2.75	2.65	24.04	2.07	10.20	27.77	39.68	14.72	26.87	32.21	47.69
**Acenaphthylene**	9.01	8.73	6.00	7.33	7.20	8.13	6.88	9.30	7.70	6.57	7.28	8.02	3.56	2.03	4.55	5.28	7.42
**Acenaphthene**	8.69	7.65	10.06	228.59	121.59	106.66	8.72	9.34	74.95	4.99	7.57	8.15	142.64	118.78	119.85	35.06	22.79
**Fluorene**	4.93	6.37	9.98	5.86	5.05	5.68	11.99	17.66	15.02	4.98	6.37	9.86	2.68	1.36	2.21	2.80	4.05
**Phenanthrene**	12.98	4.31	7.26	6.02	10.60	12.95	13.49	33.11	14.33	8.32	13.97	16.27	30.30	15.21	24.34	28.31	29.95
**Anthracene**	7.57	6.42	6.72	7.12	8.40	9.53	7.77	11.36	9.90	7.43	8.21	9.63	6.03	3.48	4.66	5.01	7.05
**Fluoranthene**	22.22	6.42	9.17	9.36	13.66	15.09	14.44	40.15	15.50	11.49	17.42	27.85	28.95	16.38	26.98	26.22	67.61
**Pyrene**	22.14	7.94	11.18	9.69	14.88	17.69	14.36	40.01	16.90	14.64	23.19	27.88	43.23	22.31	7.77	44.47	67.98
**Benz[a]anthracene**	17.09	5.28	6.04	6.19	8.11	9.66	7.71	19.64	7.50	9.91	10.92	15.19	15.21	8.89	10.06	18.86	51.08
**Chrysene**	31.95	8.56	11.79	11.89	17.18	21.65	18.42	57.66	18.24	17.02	23.36	34.24	46.07	32.70	39.95	43.00	66.31
**Benz(a)pyrene**	12.81	393.97	6.85	7.13	8.12	6.49	8.71	22.50	1.49	6.70	6.11	3.99	21.67	29.07	23.69	20.09	43.23
**Benzo[k]fluoranthene**	16.66	358.02	0.94	1.20	2.08	0.62	2.61	25.34	3.85	0.81	0.28	8.76	19.51	26.14	21.32	18.09	38.82
**indeno(1,2,3-cd)pyrene**	11.76	6.33	8.23	8.91	8.13	8.22	6.99	13.08	8.44	9.05	7.63	8.68	10.74	27.67	10.99	12.17	26.74
**Dibenz(a,h)anthracene**	5.89	6.07	5.04	5.26	5.47	4.85	5.43	6.34	5.27	5.05	4.48	4.78	2.88	8.88	3.07	3.17	7.35
**Benzo[g,h,i]perylene**	21.95	11.12	11.88	16.97	11.98	12.96	9.87	23.52	12.19	16.11	12.10	14.86	34.88	60.97	26.49	51.15	78.12
**Total**	247.85	874.38	117.04	336.59	252.98	253.12	140.12	331.67	235.32	125.15	159.06	225.93	448.03	388.57	352.79	345.88	566.19
**ΣPAH(PM2.5)/ΣPAH(PM10)**	2.58	0.73	9.44	5.10	5.67	6.58	14.57	4.52	2.51	7.40	4.47	0.59	1.04	0.69	0.97	0.98	0.88

Table S4. Diagnostic ratios of PAHs in atmospheric $PM_{2.5}$ and PM_{10} from Riyadh city, Saudi Arabia from April- December 2023.

	**4-Apr**	**25-Apr**	**10-May**	**25-May**	**10-Jun**	**25-Jun**	**10-Jul**	**25-Jul**	**10-Aug**	**25-Aug**	**10-Sep**	**25-Sep**	**10-Oct**	**25-Oct**	**10-Nov**	**25-Nov**	**11-Dec**
	**PM2.5**																
**Ratios**																	
LMW/HMW$_{(PAH)}$	0.12	0.11	0.23	0.19	0.11	0.12	0.03	0.06	0.07	0.04	0.07	0.52	0.39	0.32	1.25	0.47	0.41
Phe/Ant	1.35	0.68	0.23	1.13	1.30	1.62	2.69	2.49	1.40	1.19	1.70	1.21	5.50	1.00	5.01	5.09	4.65
Flu/Py	1.01	0.84	0.84	0.82	1.00	1.00	1.00	0.71	0.81	0.67	0.70	0.77	0.98	0.95	0.71	0.57	0.99
Ant/(Ant+Phe)	0.43	0.60	0.81	0.47	0.44	0.38	0.27	0.29	0.42	0.46	0.37	0.45	0.15	0.50	0.17	0.16	0.18
Flu/(Flu+Py)	0.50	0.46	0.46	0.45	0.50	0.50	0.50	0.41	0.45	0.40	0.41	0.44	0.50	0.49	0.41	0.36	0.50
BaA/(BaA+Chr)	0.38	0.37	0.36	0.39	0.32	0.30	0.26	0.28	0.40	0.36	0.35	0.32	0.25	0.22	0.19	0.30	0.27
Ind/(Ind+BghiP)	0.42	0.40	0.40	0.30	0.73	0.72	0.35	0.36	0.43	0.40	0.48	0.35	0.24	0.31	0.30	0.19	0.25
	**PM10**																
LMW/HMW(PAH)	0.53	0.09	0.65	3.39	1.82	1.60	0.58	0.34	1.63	0.38	0.51	0.54	1.01	0.67	1.07	0.46	0.27
Phe/Ant	1.71	0.67	1.08	0.85	1.26	1.36	1.74	2.91	1.45	1.12	1.70	1.69	5.03	4.37	5.22	5.65	4.25
Flu/Py	1.00	0.81	0.82	0.97	0.92	0.85	1.01	1.00	0.92	0.79	0.75	1.00	0.67	0.73	3.47	0.59	0.99
Ant/(Ant+Phe)	0.37	0.60	0.48	0.54	0.44	0.42	0.37	0.26	0.41	0.47	0.37	0.37	0.17	0.19	0.16	0.15	0.19
Fla/(Fla+Py)	0.50	0.45	0.45	0.49	0.48	0.46	0.50	0.50	0.48	0.44	0.43	0.50	0.40	0.42	0.78	0.37	0.50
BaA/(BaA+Chr)	0.35	0.38	0.34	0.34	0.32	0.31	0.30	0.25	0.29	0.37	0.32	0.31	0.25	0.21	0.20	0.30	0.44
Ind/(Ind+BghiP)	0.35	0.36	0.41	0.34	0.40	0.39	0.41	0.36	0.41	0.36	0.39	0.37	0.24	0.31	0.29	0.19	0.26

Figure S1. TIC of the standard mixture of polycyclic aromatic hydrocarbons (PAHs), including the followings: (1) naphthalene (Nap), (2) acenaphthylene (Acy), (3) acenaphthene (Ace), (4) fluorene (Flu), (5) phenanthrene (Phe), (6) anthracene (Ant), (7) fluoranthene (FR), (8) pyrene (Pyr), (9) benzo[a]anthracene (BaA), (10) chrysene (Chr), (11) benzo[b]fluoranthene (BbF), (13) benzo[a]pyrene (BaP), (14) indeno[1,2,3-cd]pyrene (IDP), and (15) dibenz[a,h]anthracene.

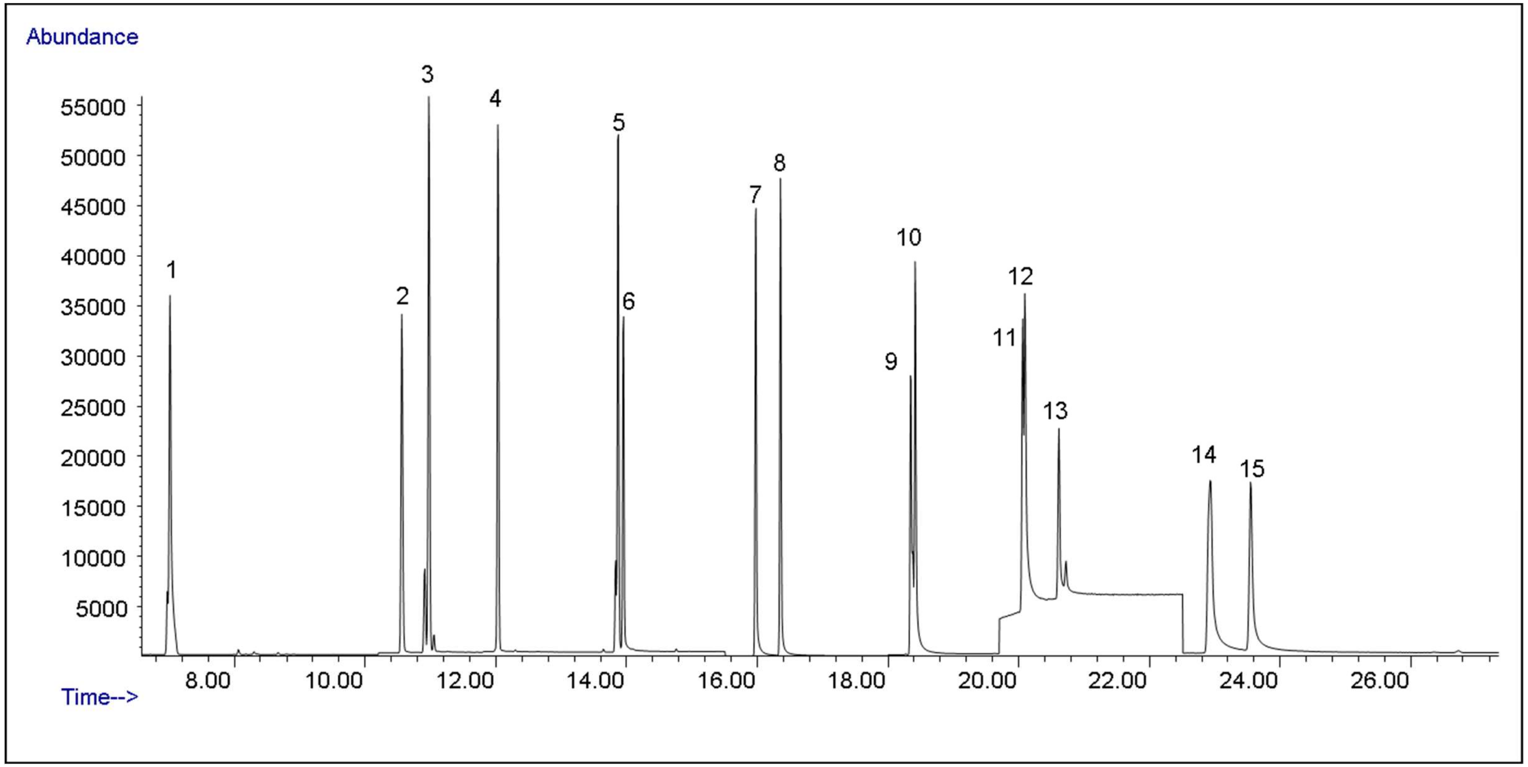

Figure S2. Plots showing (a) the concentrations, (b) the concentration ratios of $PM_{2.5}/PM_{10}$, and (c) the air quality index (AQI) of atmospheric $PM_{2.5}$ and PM_{10} from the city of Riyadh, Saudi Arabia during the moths of April-December of 2023.

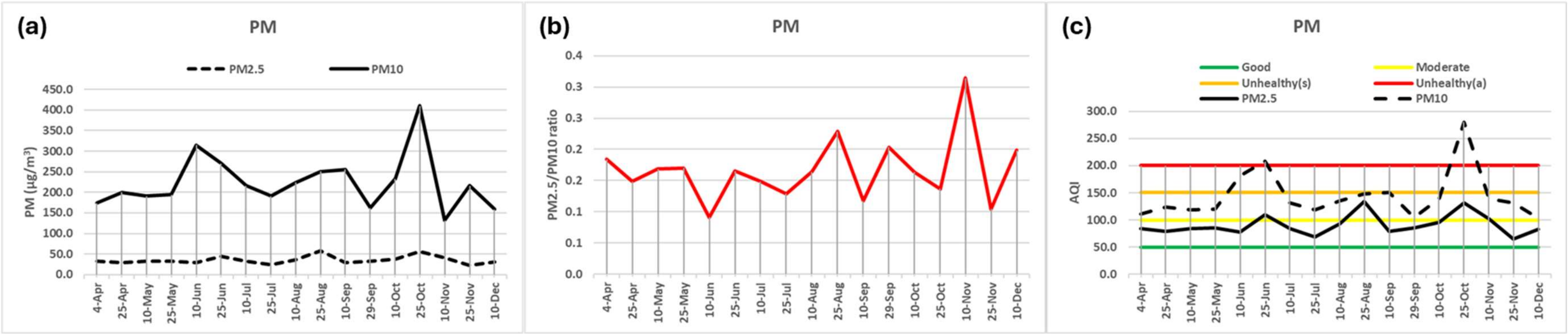

Figure S3. Box plots showing: (a) and (b) the total concentrations of PAHs in atmospheric $PM_{2.5}$ and PM_{10}, respectively and (c) the total concentration ratios of PAHs in $PM_{2.5}$ to PM_{10} from Riyadh city during the months of April-December of 2023.

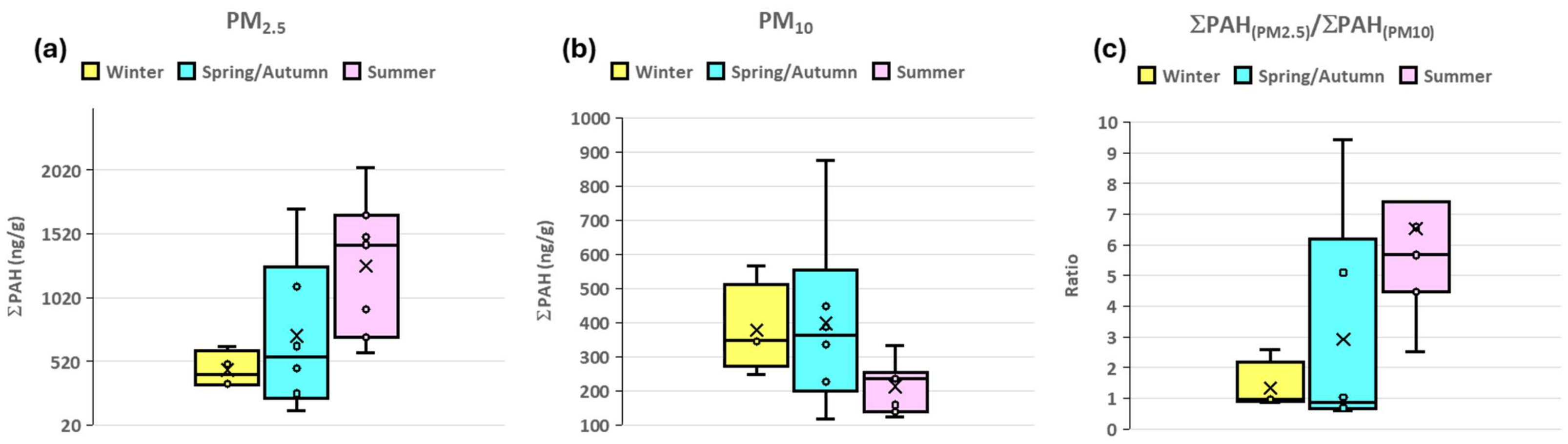

Supplement: Supplementary file 1 [file toxics-13-00424-s001.zip › toxics-3625500-supplementary.pdf]
